# Bridging success in the labour market: does continuity of general practitioners’ care matter for individuals with common mental disorders?

**DOI:** 10.1007/s10198-025-01882-4

**Published:** 2026-01-07

**Authors:** M. Kamrul Islam, Håvard Thorsen Rydland, Egil Kjerstad

**Affiliations:** Health Services & Health Economics, NORCE Health and Social Sciences, Bergen, Norway

**Keywords:** Common mental disorders (CMDs), Continuity of general practitioners’ care indices (RGP-CoCs), Labour market outcomes, Employment, Sickness absence, Longitudinal study, Administrative registries, Norway, I12, I14, J21, C24

## Abstract

Mental illnesses impose substantial burdens on individuals, families, and society, encompassing both severe personal consequences and high societal costs. This study examines whether improved continuity of care with regular general practitioners (RGP-CoC) is associated with better labour market outcomes for individuals diagnosed with common mental disorders (CMDs). Using administrative registry data for 139,873 individuals with CMDs (*N* = 371,825 observations) from 2014/15 to 2017/18, we construct RGP-CoC indices within rolling two-year windows and track labour market outcomes over subsequent one-year periods. We employ a lagged design and high-dimensional fixed-effects models to robustly assess the association between RGP-CoC and labour market outcomes over the period 2016–2019. Our findings show that higher RGP-CoC is associated with improved labour market outcomes, with stronger and more robust effects observed for wage income than for employment probability. We also find a significant negative association between RGP-CoC and the likelihood of sickness absence. While the effects vary across education levels, no significant gender differences are observed. These findings highlight the vital role of RGP continuity of care in improving labour market participation and earnings, especially for individuals with CMDs. Enhancing continuity with a regular GP as a core quality metric in primary care can inform healthcare policy and support broader goals of economic inclusion.

**Supplementary Information:**

The online version contains supplementary material available at 10.1007/s10198-025-01882-4.

## Introduction

The detrimental effects of mental illness on individuals, families and society can be severe, both in terms of well-being and work-related costs. According to the World Health Organization (WHO) common mental disorders (CMDs) encompass two primary diagnostic categories: depressive disorders and anxiety disorders. These conditions show high prevalence across the world population [1]. According to OECD, CMDs such as depression, anxiety disorders and alcohol and drug use disorders, affect more than one in six people across the European Union in any given year. Besides the impact on people’s health and well-being, the report estimates the total costs of mental ill-health at over EUR 600 billion – or more than 4% of GDP – across the 28 EU countries [2]. A large part of these costs is due to lower employment rates and lower productivity. Studies find that people with CMDs have adverse labour market outcomes than people without any mental disorders (for details, see [3]). CMDs among the working-age population is a critical concern for labour market and social policies across several OECD countries [4].

CMDs can be associated to labour market outcomes through multiple pathways. However, causal analyses of the relationship between CMD and labour market outcomes are complicated because of omitted variable bias and reverse causality due to potential endogeneity of CMDs. Omitted variable bias arises due to “third” factors (e.g., genetic endowments, cognitive ability, childhood circumstances, personality) that are correlated with both CMDs and labour market outcomes, while reverse causality occurs when lack of employment or reduced earnings worsen mental health [5]. Endogeneity concerning CMDs and labour market outcomes are analytically challenging. It is difficult to perform unbiased estimation of the impact of CMDs on labour market outcomes, i.e., to compare the labour market performance differences between healthy individuals and people with CMDs. Having such complexities in mind, our point of departure here is to focus on whether quality primary care is associated with labour market attachment for people with CMDs.

Continuity of care (CoC) has been considered a core value of primary care services [6, 7] and as such represents an important indicator of the quality of services provided^1^. Recent research shows that higher level of continuity of regular general practitioners (RGPs) care is associated with reduced use of emergency and specialist health care [9–13] with improved compliance with medication prescriptions [14], and lower mortality [15, 16]. Evidence also suggests that continuity with RGPs care is strongly associated with greater patient satisfaction with care (e.g., [17]. A recent registry-based observational study in Norway also echoed similar findings – continuity of GP care is significantly associated with lower mortality, fewer acute hospital admissions and lower use of emergency (out-of-hours) services [11].

This study considers relational continuity of care (CoC) with individuals’ RGPs [16]. Our hypothesis is that CoC with individuals RGPs (RGP-CoC) as a quality indicator of primary care extends beyond GP’s technical proficiency, correspondingly encompassing compassion and commitment that foster trust between patients and physicians nurtured through shared experiences [18, 19]. Within this framework, RGP-CoC can be a manifestation of cognitive social capital and can be operationalised through individuals’ perception of the level of interpersonal trust [20]. Therefore, we propose that better RGP-CoC may not only improve health outcomes and reduce healthcare costs [21, 22] but also have a beneficial spill-over effects on patients’ labour market outcomes. RGP-CoC may foster a trusting relationship between patients and their RGPs, leading to improved emotional support and guidance. Through such a mechanism the patients with CMDs feel supported and understood by their RGPs reducing stress levels, improve coping mechanisms, and increase resilience, all of which can contribute to better performance in the labour market [23, 24]. Moreover, by addressing CMD symptoms early and by providing consistent and proactive management of mental health conditions, better RGP-CoC may help prevent work disruptions due to symptom exacerbations or crises [25, 26]. We also argue that better RGP-CoC can help individuals to maintain stable employment and avoid sickness absences or job loss associated with untreated or poorly managed mental health conditions. In summary, the mechanisms through which better RGP-CoC predicts labour market outcomes for individuals with CMDs involve a combination of improved health and functioning, enhanced well-being and resilience, access to supportive services, and prevention of work disruptions, all of which contribute to greater participation in the labour market.

Despite growing recognition of the importance of continuity in healthcare, there is a notable lack of research examining the effects of continuity of GP care on labour market outcomes, particularly among individuals with common mental disorders (CMDs). Existing studies have largely focused on health outcomes or healthcare utilization, leaving a gap in understanding how sustained relationships with GPs may influence economic participation. So far, few studies consider the effect of CoC on labour market outcomes. For example, Sheehan et al. [27] have studied the association of provider CoC with work time loss among patients with low back pain and found that higher CoC is associated with lower working time loss.

This study aims to assess whether continuity of care with regular general practitioners (RGP-CoC) is associated with improved labour market attachment among individuals diagnosed with CMDs. Using comprehensive Norwegian registry data, our analytical approach uses a panel data framework with worker, GP, and municipality fixed effects. We construct a longitudinal dataset of 139,873 individuals with CMDs and follow their interactions with GPs over multiple time periods. CoC is measured over a two-year baseline period, followed by a one-year window for observing labour market outcomes. This lagged design with fixed-effects approach helps mitigate reverse causality—where poor labour market outcomes or health could influence GP visits—and allows for a more accurate estimation of CoC’s effect on subsequent labour market outcomes. Our findings show that higher levels of RGP-CoC are linked to better labour market outcomes—especially higher wage income—and a reduced likelihood of sickness absence.

This study contributes to the literature by being among the first to rigorously examine the association between GP-CoC and labour market outcomes using nationally representative, longitudinal data. Existing research is limited in both scope and methodology, often relying on cross-sectional designs or focusing solely on health-related endpoints. By incorporating time-varying GP-CoC measures, robust fixed-effects models, and focusing on an economically relevant population—individuals with CMDs—we address key gaps in the literature and offer new insights into the potential of continuity of care as a policy lever to improve not just health, but also economic inclusion and productivity.

The rest of the paper is organized as follows: The Institutional Setting and Data section provides an overview of key features of the Norwegian regular GP Scheme. The Data and Definition of Variables subsection describes the data sources, inclusion criteria, and variables used in the analysis. The Analytical approach section details our estimation methods. The Results section presents descriptive statistics, main findings, and additional heterogeneity analyses. The final section discusses the results and concludes the paper.

## Institutional setting

In Norway, GPs operate within a decentralized healthcare system where municipalities are responsible for organizing and funding primary care services. The system is structured around the “Regular GP Scheme” (fastlegeordningen), introduced in 2001, to improve access, continuity, and quality of primary care. Under this system, all residents have the right to register with a regular GP of their choice, as long as the GP has capacity on their patient list. Each GP maintains a personal GPlist—a roster of individuals for whom they are responsible. The average list contains about 1,100–1,200 patients, though this can vary by region and GP availability [28]. Patients can switch GPs up to twice per year through the Norwegian Health Economics Administration (HELFO) portal. This structure supports long-term doctor–patient relationships, encourages preventive care, and provides stability in care delivery.

Most GPs are self-employed and work under contractual agreements with municipalities. They run their own practices, manage their own patient lists, and are reimbursed through a mix of capitation (per patient per year), fee-for-service payments (per consultation or procedure), and patient co-payments. Around 10% GPs are salaried employees of the municipality.

GPs often work in group practices, where several general practitioners share clinic space, administrative staff, and sometimes medical equipment. While each GP maintains their own patient list (GPlist), the shared environment allows for collaboration and cross-coverage when individual doctors are absent due to illness, vacation, or training. This setup can support CoC by ensuring that patients still have access to medical services from familiar colleagues within the same practice when their regular GP is unavailable. In addition, group practices often include nurses or allied health professionals, allowing for more comprehensive care coordination. Group practices typically consist of 2–6 doctors, but larger centres exist in urban areas. These practices often include other healthcare professionals like nurses, physiotherapists, or midwives, fostering a more team-based approach to primary care.

Primary care outside of regular GP hours — during evenings, nights, weekends, and public holidays — is provided through a system known as the out-of-hours (OOH) emergency primary care service, or Legevakt. This service is organized at the municipal or inter-municipal level and is designed to handle urgent, but not life-threatening, medical situations that cannot wait until regular GP offices reopen. Services may include in-person consultations, telephone advice, and home visits in special cases. For life-threatening emergencies, patients are instructed to call 113 (the emergency medical service). While the Legevakt system ensures access to care 24/7, it can pose challenges in terms of continuity, since patients are often treated by doctors other than their regular GP.

## Data and methods

Our data extracted from administrative registries covering the entire population of Norway. Individuals’ socio-demographic characteristics, education, wage income, and sickness absence data are gathered from Statistics Norway (SSB). Information on GP visits comes from the database Control and Reimbursement of Health Care Claims (the KUHR registry). The dataset has information on individuals GP visits, either to their regular GP or a GP in the same group practice during regular opening hours or visits to emergency out-of-hours (OOH) unit. The data originates from reimbursement claims sent by the primary care physician (after each consultation). With the inclusion of individual identifiers and consultation dates in the KUHR registry, we are able to track the number of primary physician visits for each individual within a specific period. The KUHR data also has GP identifiers that is merged with information from the regular GP database with patient list information and GP characteristics (see Table 1). This enables us to verify whether the visit was with the individual’s regular GP, another GP, or during OOH care.

**Table 1:** Definitions and descriptive statistics of variables used in the analysis (2016–2019)

Variable	All year (*N* = 371,825)	2016 (*n* = 93,904)	2017 (*n* = 109,549)	2018 (*n* = 95,400)	2019 (*n* = 72, 972)
	Mean/Percent (Std. dev.)	Mean/Percent (Std. dev.)	Mean/Percent (Std. dev.)	Mean/Percent (Std. dev.)	Mean/Percent (Std. dev.)
Employment Probability (%)	74.9 (43.3)	74.3 (43.7)	75.3 (43.2)	75.9 (42.8)	74.1 (43.8)
Wage income (in NOK)	293,378 (291,946)	265,477 (270,803)	291,420 (285,588)	310,728 (305,210)	309,542 (306,663)
Sickness absence probability (%)	46.9 (49.9)	51.2 (50.0)	45.9 (49.8)	46.8 (49.9)	42.9 (49.5)
Amount received for sickness compensation (in NOK)	29,490 (68,011)	31,871 68,861	28,523 (66,880)	29,181 (67,901)	28, 282 (67,901)
UPC index	0.835 (0.304)	0.837 (0.296)	0.837 (0.300)	0.843 (0.297)	0.818 (0.327)
CoC-GP index	0.826 (0.178)	0.818 (0.180)	0.821 (0.185)	0.831 (0.175)	0.834 (0.169)
Workers gender-male(%)	37.2 (48.3)	37.1 (48.3)	37.5 (48.4)	37.3 (48.49	36.9 (48.2)
Whether individual use specialist physical care (spe_Phy)	0.631 (0.483)	0.633 (0.482)	0.629 (0.483)	0.629 (0.483)	0.635 (0.481)
Whether individual use specialist phycological care (Spe_Psy)	0.002 (0.044)	0.002 (0.048)	0.002 (0.045)	0.002 (0.043)	0.001 (0.039)
Low Education (Educ1: omitted category)	0.265 (0.441)	0.283 (0.450)	0.266 (0.442)	0.254 (0.435)	0.254 (0.436)
High School Education (Educ2)	0.337 (0.473)	0.339 (0.473)	0.340 (0.474)	0.336 (0.472)	0.332 (0.471)
Short higher education (Educ3)	0.318 (0.466)	0.306 (0.461)	0.316 (0.465)	0.325 (0.468)	0.328 (0.469)
Long higher education (Educ4)	0.080 (0.271)	0.071 (0.258)	0.079 (0.269)	0.085 (0.279)	0.085 (0.279)
GP-characteristics					
Whether GP practicing in a group (GP_group)	0.914 (0.280)	0.912 (0.283)	0.914 (0.281)	0.916 (0.278)	0.916 (0.277)
Whether GP has open list to take new patient (GP_open)	0.207 (0.405)	0.247 (0.431)	0.213 (0.409)	0.191 (0.393)	0.168 (0.374)

Information on use of specialist psychological and physical healthcare services—-inpatient and outpatient—was gathered from the Norwegian Patient Register (NPR) database. Private hospitals and outpatient specialists primarily deliver elective treatments, usually under contractual agreements with public health authorities. This information is also recorded in the NPR database.

### Sample selection

Our study included individuals born between 1959 and 1990, corresponding to ages 24 to 55 in 2014, with the maximum age reaching 60 by 2019. This inclusion criteria had ensured us to construct a homogeneous group of individuals with CMDs. The lower age bound was chosen on the ground that free high school education in Norway is available until a person turns 24 years of age. The upper age bound is chosen since individuals reaching 62 years of age, are eligible to claim early retirement pensions.

We further restricted our sample to individuals diagnosed in primary care with one or more of the CMDs according to the International Classification of Primary Care, 2nd edition (ICPC-2) codes. The included CMDs are— anxiety, depression, stress, insomnia, substance abuse, and other common mental disorders (CMDs) (see, e.g., [29]).

To ensure consistent exposure to the same RGP, we included only patients who remained registered with the same GP–patient list during the baseline period. The baseline period is defined as consecutive two-year intervals—2014–2015, 2015–2016, 2016–2017, and 2017–2018—prior to measuring labour market outcomes in 2016, 2017, 2018, and 2019, respectively. For each period, we include only GPs who were practising as of January 1 of the first year of that baseline period (i.e., January 1, 2014; January 1, 2015; January 1, 2016; and January 1, 2017, respectively). This approach allows us to identify a unique preferred regular GP for each patient and construct a consistent measure of CoC.

The next inclusion criterion was that each patient remain registered with the same GP–patient list throughout the respective baseline period. This ensured that a unique preferred GP could be identified for each included patient. We excluded individuals who changed GPs during the baseline period for key reasons. Our focus is on CoC with the same GP, which reflects an ongoing therapeutic relationship. Patients who switch GPs may do so for reasons unrelated to care quality, complicating the measurement and interpretation of CoC. By restricting the sample to patients with the same GP, we better capture individual-level relational continuity—the sustained, personal relationship between a patient and their GP. This restriction enhances comparability across patients and strengthens the internal validity of our findings. Finally, following the earlier studies on the measures of continuity, we also restricted the sample to individuals who had at least two consultations with primary care physicians during the baseline period (e.g., [9]). With these inclusion criteria our final sample formed a total of 139,873 individuals with CMDs (*N* = 371,825) enlisted to 2,683 GPs and lived in 422 different municipalities.

### Dependent variables

#### Employment probability and wage income

Employment probability is defined by whether an individual has participated in the labour market during our study period and the outcome is operationalized by whether an individual had a positive wage income or not (yes/no).

*Wage income* is defined as earnings from employment only and excludes sickness absence benefits. To reduce skewness, we use the logarithm of wage income as the dependent variable, treating it as a continuous measure.

#### Sickness absence probability

*Sickness absence probability* is defined by whether an individual has received doctor-certified sickness absences from work, operationalised by whether an individual received compensation for loss of income due to illness or injury (yes/no).

### Independent variables

#### RGP-CoC measures

Two alternative measures were used. The first, the Usual Provider of Care (UPC) index, is a widely used indicator of continuity of care, capturing the extent to which a patient consistently consults the same healthcare provider (i.e., RGP) over time. It is calculated as the proportion of a patient’s total visits that are made to their most frequently seen provider [10], and can be defined as: $\:UPC=\frac{{V}_{g*}^{i}}{\sum\:_{g=1}^{G}{V}_{g}^{i}}$ where, numerator $\:{\:V}_{g*}^{i}$ is number of visits by an individual patient *i* to his own regular/*registered* GP, g* and the denominator implies the total number of visits to all primary care providers ($\:{V}_{g}^{i}$) contacts during a given period. For example, if an individual has 10 contacts in total, and 7 of them are with his own RGP, the UPC index will be 0.7. The index ranges from 0 to 1, where a value of 1 indicates perfect continuity (all visits were with the own RGP), and lower values suggest that care was spread across multiple primary care providers.

Hetlevik et al. [9] critically argued that the conventional UPC index may may fail to account for unobserved health conditions For instance, patients may require acute medical care, prompting them to consult a GP other than their RGP. Moreover, patients may prioritize rapid access to any available provider over continuity with a specific one. Such preferences can affect relational CoC, as measured by the UPC index, independently of the CoC actually provided at the GP service level. These considerations led us to incorporate the alternative CoC index proposed by Hetlevik and colleagues [9]. This proposed index is based on the CoC experienced by patients other than the individual in question but who are registered with the same GP. For each patient *i*, the index uses information on the continuity experienced by other patients on the same GP’s list. Formally, the index can be defined as: CoC-GP$\:=\frac{{V}_{g*}^{-i}}{\sum\:_{g=1}^{G}{V}_{g}^{-i}}$; where *i* is an individual, *g* is one of *G* GPs, g* is the GP whom individual *i* is enlisted with and $\:{V}_{g}^{-i}$is the number of visits to GP *g* of all individuals enlisted with g*, except for *i* [9]. As exemplified by Hetlevik et al. [9], consider three patients—A, B, and C—enlisted with the same GP. If A has 8 out of 10 visits with their RGP, B has 3 out of 7, and C has 12 out of 12, then the CoC-GP index is calculated as: A’s score = (3 + 12)/(7 + 12); B’s score = (8 + 12)/(10 + 12); C’s score = (8 + 3)/(10 + 7). This approach produces an index value between 0 and 1, where a higher score indicates greater CoC within the patient’s GP list population.

It is unlikely that the relationship between continuity of GP care and labour market outcomes is strictly linear. To account for potential non-linearities, we include quadratic and cubic terms of the CoC indices in the model. This approach allows us to capture more complex patterns, such as diminishing or increasing marginal effects, thereby improving model fit and reducing the risk of misspecification when the impact of continuity of GPs care varies across its range.

### Other covariates

#### Individual level covariates

Health status is proxied by two indicators—whether an individual used specialist psychiatric healthcare (yes/no), and specialist somatic healthcare services (yes/no) during baseline period. “No” is considered as the omitted category.

Education qualifications were classified into four groups: (i) Compulsory education or lower (low-educated individuals, the omitted category); (ii) High school education; (iii) First stage (undergraduate) higher education, and (iv) Second stage (postgraduate) higher education.

#### GP level covariates

GP-level time-varying covariates comprise the practice setting (group vs. solo) and the status of the patient list (open vs. closed, i.e., whether it has vacancies for new patients). (see Table 1).

### Analytical approach

We use a prospective longitudinal study design at the individual level. Our empirical strategy uses a two-year baseline period to measure RGP-CoC indices, followed by a one-year period in which labour market outcomes are observed. Within this framework, the RGP-CoC indices are calculated over consecutive two-year baseline periods—2014–2015, 2015–2016, 2016–2017, and 2017–2018. We then assess the associations between these time-varying RGP-CoC indices and individuals’ labour market outcomes measured annually in the subsequent years—2016, 2017, 2018, and 2019, respectively. This design helps mitigate potential reverse causality: poor labour market outcomes or worsening health may increase GP visits, which could either inflate CoC indices (if visits are with the RGP) or deflate it (if with other GPs). By lagging the measurement of CoC indices relative to labour market outcomes, we reduce potential bias and more accurately estimate the effect of RGP-CoC on subsequent labour market outcomes.

Potential endogeneity between RGP-CoC and labour market outcomes may arise due to unobserved factors influencing both. To address this, we employ a panel data approach with worker fixed effects to control for time-invariant individual characteristics. To reduce bias due to sample heterogeneity, our models are also adjusted for a series of individual-level covariates (X) that may influence the association between labour market outcomes and RGP-CoC, namely, education and initial health status indicators, as detailed above.

In our models, the time fixed effects control for underlying observable and unobservable systematic differences over calendar time. We further included GP fixed effects, which control for unobserved, time-invariant characteristics specific to each GP, such as practice style, quality of care, or patient management approach. By including these fixed effects, we isolate the impact of CoC on labour market outcomes from GP-level differences, thereby reducing potential bias caused by systematic variations between GPs. This approach ensures that our estimates more accurately reflect the effects of RGP-CoC itself, rather than confounding factors related to individual GPs.

Additionally, our models included municipality-fixed effects (where the GP practices are based). The municipality-fixed effects estimator adjusts for any time-invariant differences between municipalities, including factors like centrality and availability of specialist health services.

The explicit introduction of dummy variables is not a feasible option to account simultaneously for these four sources of time invariant unobserved heterogeneity (i.e., the fixed-effects)–the worker, GP, municipality and time–because the number of units of workers, GPs, and municipalities are too large. Therefore, we use the following high-dimensional fixed-effect model [30]^2^ :

1 $$\begin{aligned}&\\&\:{l}_{igmt}={X}_{igmt}\beta\:+{\theta\:}_{gt}\delta\:+&\\&{\nu\:}_{i}+{\psi\:}_{g}+{\lambda\:}_{m}+{\delta\:}_{t}+{\epsilon\:}_{igmt}\end{aligned}$$,

where the dependent variable $\:{l}_{igmt}$represents the labour market outcome for individual *i* with registered GP *g*, in municipality *m* and year *t*. For binary outcomes, $\:{l}_{igmt}$​ equals 1 if the individual has positive wage income or has received doctor-certified sickness absence, and 0 otherwise. For continuous outcomes, $\:{l}_{igmt}$ indicates the logarithm of wage income.

The vector$\:{\:X}_{igmt}$​ includes observed time-varying characteristics of the individual, while $\:{\theta\:}_{gt}$​ captures time-varying characteristics of the GP. The term $\:{\nu\:}_{i}$denotes individual fixed effects, $\:{\psi\:}_{g}$ indicates GP fixed effects, $\:{\lambda\:}_{m}$accounts for municipality-level fixed-effects, $\:{\delta\:}_{t}$​ includes time fixed effects common to all individuals and municipalities, and $\:{\epsilon\:}_{igmt}$represents the idiosyncratic error term.

We select the functional form of our main dependent variable, the CoC index—whether linear, quadratic, or cubic—based on model fit criteria, specifically the Akaike Information Criterion (AIC) and the Bayesian Information Criterion (BIC). We choose the model with the lowest AIC and/or BIC values among the competing models. These criteria enable us to compare alternative model specifications and select the one that best balances goodness of fit with model complexity, since the marginal effect of no-linear independent variable (e.g. RGP-CoC index) varies depending on its value. We report the Average Marginal Effect (AME) to provide a clear and interpretable summary of how, on average, changes in the CoC index influence the labour market outcomes. To obtain the AME, we calculate the derivative of Eq. 1 with respect to the CoC index at each observed value, and then average these effects across the sample.

To account for potential correlation of error terms within workers over time and within GPs and municipalities due to shared local factors, we cluster standard errors at both the individual, GP and municipality levels. The individual-level clustering addresses serial correlation from repeated observations of the same person across multiple years, while GP and municipality-level clustering accounts for unobserved heterogeneity and spatial correlation in labour market conditions and healthcare access. This approach ensures robust inference by addressing both serial and spatial correlations in the data.

## Results

### Descriptive statistics

**Fig. 1:**
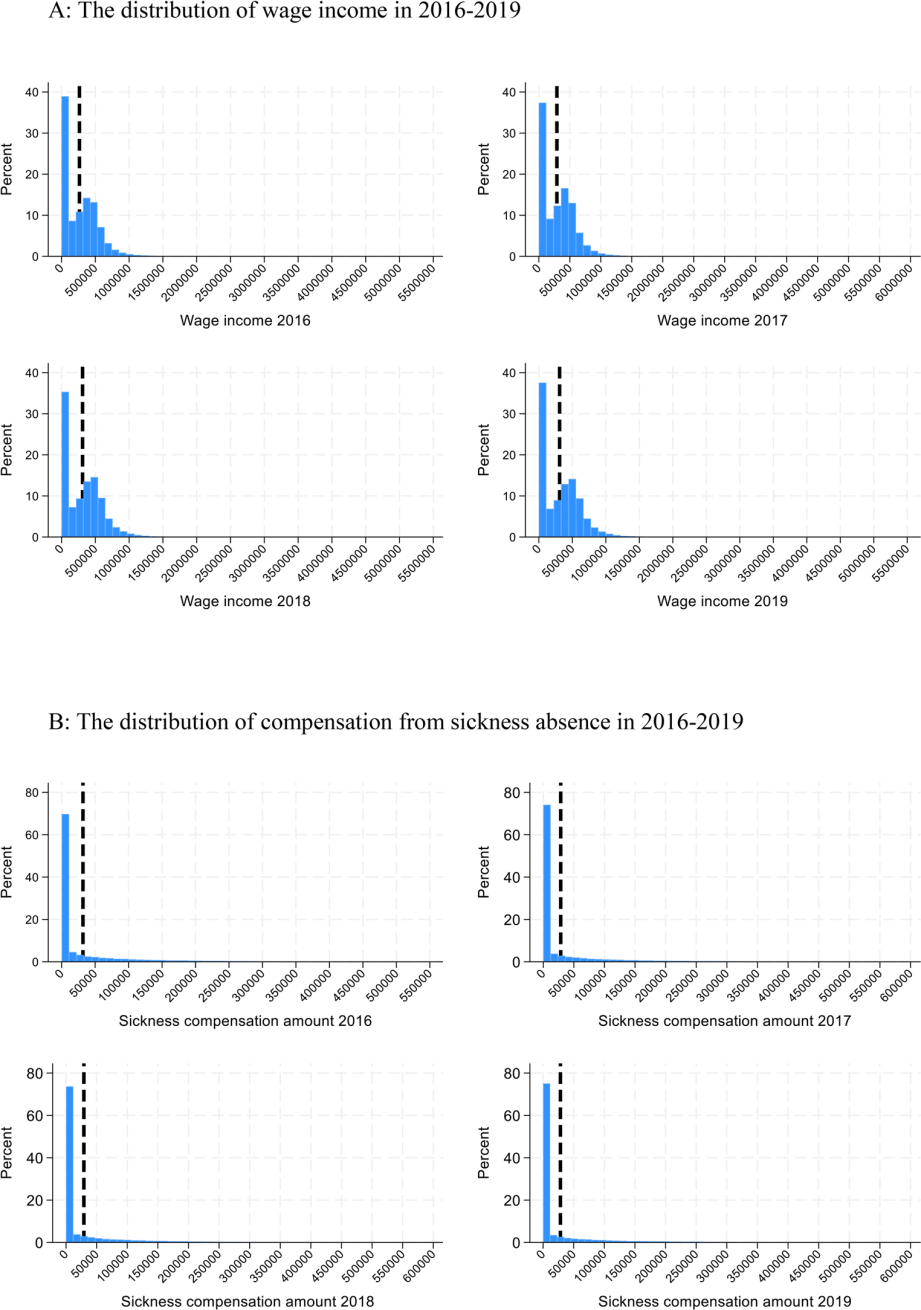
**A**: The distribution of wage income in 2016-2019 **B**: The distribution of compensation from sickness absence in 2016-2019

Table 1 provides summary statistics for the full sample across the years 2016–2019, comprising a total of 371,825 observations. The average employment probability over-all years is 74.9%, remaining relatively stable across years. Average annual wage income (in NOK) increased from 265,477 in 2016 to approximately 309,500 in 2019, reflecting general income growth over time. The sickness absence probability was 46.9% overall, with a notable decline from 51.2% in 2016 to 42.9% in 2019. The distributions of the two labour market outcomes are presented in Fig. 1A and B. Both dependent variables exhibit pronounced skewness: approximately 24–25% of individuals report zero wage income over the years, while 49–57% of the sample report no sickness absence during the same period.

**Fig. 2:**
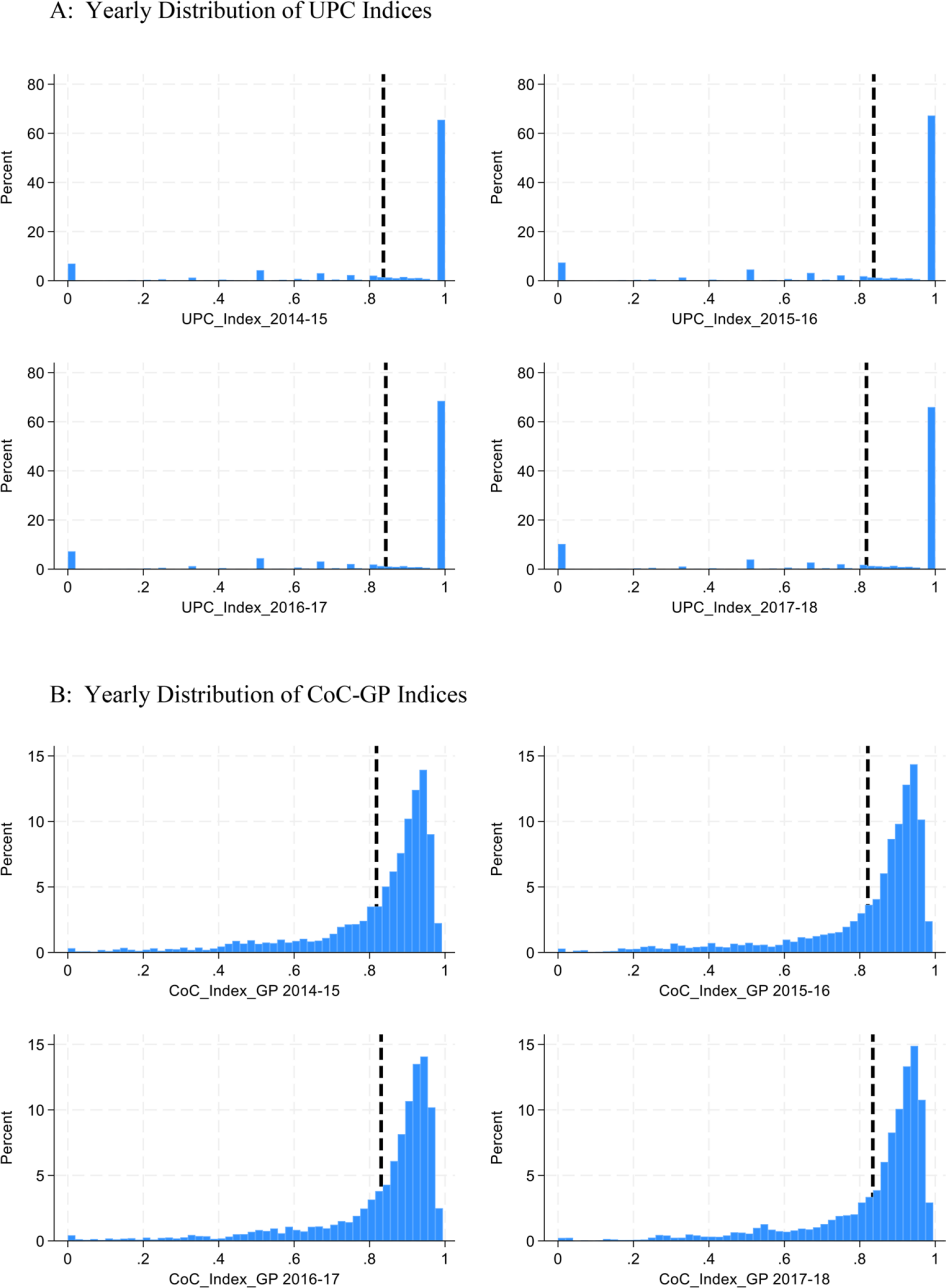
**A**: Yearly distribution of UPC indices **B**: Yearly distribution of CoC-GP indices

The UPC index averaged 0.835, and the CoC-GP index averaged 0.826, both indicating relatively high continuity of care. These measures were stable across years with only minor fluctuations. The distributions of the two CoC indices are shown in Fig. 2A and B. The UPC index exhibits highly discontinuous variation: over the years, more than 60% of the sample had an index value of one, indicating that all GP visits during the baseline periods were with their own GP, while 7–10% had no visits to their own GP. In contrast, the CoC-GP index displays a continuous but strongly right-skewed distribution across all years.

37.2% of individuals were male, and the use of specialist physical care services was reported for 63.1% of individuals. The use of specialist psychological care was rare, reported by only 0.2% of the sample. Educational attainment shows that 26.5% had low education (the omitted reference category), 33.7% had completed high school, 31.8% had short higher education, and 8% had long higher education. The distribution of education levels was relatively stable across years.

Regarding GP characteristics, 91.4% of GPs practiced in group settings. The proportion of GPs with an open list to accept new patients decreased from 24.7% in 2016 to 16.8% in 2019.

### Regression results

Table 2 presents the results from fixed-effects regressions estimating the association between two RGP-CoC indices and two key labour market outcomes: employment probability and log wage income. To account for potential non-linear relationships, the models include quadratic and cubic terms for the RGP-CoC indices. As mentioned, these specifications were selected based on model fit criteria, specifically the AIC and the BIC values. Given that marginal effects in non-linear models are not constant and may vary depending on the values of other covariates and the specific point on the curve, we report average marginal effects (AMEs) to summarise the association between the CoC indices and the probability of employment and wage outcomes.

**Table 2:** Main estimation results from fixed-effects models: associations between CoC indices and labour market outcomes (Employment probability and wage income)

Variable	Dependent variable: Employment probability		Dependent variable: Log of Wage Income	
	UPC Index	CoC-GP index	UPC Index	CoC-GP index
	Coefficient (Robust std. err.)	Coefficient (Robust std. err.)	Coefficient Robust std. err.	Coefficient Robust std. err.
Linear CoC	−0.022^**^ (0.011)	0.040 (0.042)	0.208 (0.086)	0.411 (0.157)
Quadratic (CoC^2^)	0.015^*^ (0.009)	−0.115 (0.077)	−0.858 (0.191)	−1.182 (0.339)
Cubic (CoC^3^)		0.080^*^ (0.045)	0.648 (0.119)	0.828 (0.211)
Average marginal effect (AME) of CoC	**0.003** **(0.005)**	0.021^*^ **(0.012)**	0.314^***^ **(0.045)**	0.232^***^ **(0.054)**
Number of observations	*N* = 371,825	*N* = 371,825	*N* = 269,680	*N* = 269,680
Workers fixed effects (number of clusters)	Yes (*n* = 139,873)	Yes (*n* = 139,873)	Yes (*n* = 105,800)	Yes (*n* = 105,800)
GP fixed effects (number of clusters)	Yes (*n* = 2,683)	Yes (*n* = 2,683)	Yes (*n* = 2,588)	Yes (*n* = 2,588)
Municipality Fixed effect (number of clusters)	Yes (*n* = 422)	Yes (*n* = 422)	Yes (*n* = 420)	Yes (*n* = 420)
Time fixed effects (number of clusters)	Yes (*n* = 4)	Yes (*n* = 4)	Yes (*n* = 4)	Yes (*n* = 4)
Adjusted R^2^	0.762	0.762	0.689	0.689

Std. Err. adjusted for clustering at worker, GP and municipality levels

All models are also control for individual and GP level time-varying covariates describe in Table 1

*, **and *** indicate the statistical significance level at the 10, 5 and 1% level

The table presents estimates for both the UPC index and the CoC-GP index. All models include fixed effects at the worker, general practitioner, municipality, and year levels to control for time-invariant individual characteristics and contextual factors.

For employment probability, the results suggest that higher RGP-CoC is associated with a modest increase in the likelihood of being employed, though the magnitude and statistical significance differ between the two indices. The AME for the UPC index is small and statistically insignificant (AME = 0.003, SE = 0.005), suggesting that patient-level continuity may not have a meaningful impact on employment probability for the individuals with CMDs. In contrast, the CoC-GP index shows a positive and weakly statistically significant AME at the 10% level. (AME = 0.021, SE = 0.012). This indicates that continuity of GP care measured at the GP level—reflecting the broader consistency of care provided to all patients by a GP—is more strongly associated with employment outcomes than individual-level continuity alone.

For wage income, the association with RGP-CoC appears stronger and more robust. Both RGP-CoC indices are positively and significantly associated with log wage income. The average marginal effect (AME) for the UPC index is 0.315 (SE = 0.045), while the AME for the CoC-GP index is 0.233 (SE = 0.054), with both effects statistically significant at the 1% level. These findings suggest that higher continuity of care—whether measured at the individual or GP level—is associated with higher earnings in the following year. Specifically, a 10% point increase in the UPC index is associated with an average 3.15% increase in wage income, while the same increase in the CoC-GP index corresponds to a 2.33% increase. The relatively stronger association observed for the UPC index may reflect the more direct relational aspects of continuity experienced at the individual level, although both measures capture meaningful and economically relevant effects.

The inclusion of quadratic and cubic terms further suggests that the relationship between CoC and labour market outcomes is non-linear. In particular, the statistically significant cubic terms in the CoC-GP models imply the presence of threshold or diminishing returns effects—where RGP-CoC is beneficial up to a point, beyond which the marginal gains may taper off or even reverse slightly. This is important for policy interpretation, as it indicates that the benefits of RGP-CoC may not be uniformly distributed across all levels of continuity.

Table 3 presents the main estimation results examining the association between RGP-CoC indices and sickness absence probability. The models are also estimated using both the UPC index and CoC-GP index. In line with previous analyses, the models include linear, quadratic, and cubic terms to account for potential nonlinearities in the relationship between RGP-CoC and outcome.

**Table 3:** Main estimation results from fixed-effects models: associations between CoC indices and sickness absence probability

Variable	Dependent variable: Sickness Absence probability	
	UPC Index	CoC-GP index
	Coefficient (Robust std. err.)	Coefficient (Robust std. err.)
Linear CoC	−0.277^***^ (0.038)	−0.301^***^ (0.089)
Quadratic (CoC^2^)	0.879^***^ (0.092)	0.798^***^ (0.169)
Cubic (CoC^3^)	−0.632^***^ (0.057)	−0.540^***^ (0.099)
Average marginal effect (AME) of CoC	−0.307^***^ **(0.019)**	−0.139^***^ **(0.023)**
Number of observations	*N* = 371,825	*N* = 371,825
Workers fixed effects (number of clusters)	Yes (*n* = 139,873)	Yes (*n* = 139,873)
GP fixed effects (number of clusters)	Yes (*n* = 2,683)	Yes (*n* = 2,683)
Municipality Fixed effect (number of clusters)	Yes (*n* = 422)	Yes (*n* = 422)
Time fixed effects (number of clusters)	Yes (*n* = 4)	Yes (*n* = 4)
Adjusted R^2^	0.511	0.511

Std. Err. adjusted for clustering at worker, GP and municipality levels

All models are also control for individual and GP level time-varying covariates describe in Table 1

*, **and *** indicate the statistical significance level at the 10, 5 and 1% level

The results show a strong negative association between the CoC indices and the likelihood of being on sickness absence. For both indices, the linear, quadratic, and cubic terms are statistically significant, indicating a nonlinear relationship. The AME for the UPC index is − 0.307 (SE = 0.019), while for the CoC-GP index it is − 0.140 (SE = 0.023). These findings imply that higher RGP-CoC is associated with a lower probability of sickness absence. Specifically, a 10% point increase in the UPC index corresponds to an average 3.07% decrease in the probability of being on sickness absence, while the same increase in the CoC-GP index corresponds to a 1.4% decrease. Similar to the relationship with wage income, the effect on sickness absence probability is more pronounced when continuity is measured at the individual level rather than at the GP level.

Detailed regression results are presented in Tables A1 and A2 in the Appendix. As expected, poor health—proxied by the use of specialist care—is negatively associated with employment and wage income, and positively associated with the likelihood of sickness absence. Consistent with prior expectations, higher levels of education are positively associated with both employment probability and wage income. However, somewhat unexpectedly, higher education is also associated with a greater probability of sickness absence. This may reflect increased health awareness, better access to benefits, or a greater likelihood of seeking medical certification among more educated individuals. In contrast, characteristics of GPs show no significant association with any of the individual labour market outcomes.

### Heterogeneity

#### Education

While factors like income, occupation, and neighbourhood can all indicate socioeconomic status, education is widely recognized as one of the most stable and foundational indicators of SES—particularly in population-based health and labour market research. Education may have different implications for CMD-diagnosed patients, and there may be heterogeneity in the relationships between RGP-CoC indices and their labour market prospects. Education shapes individuals’ access to information, health literacy, and navigation of healthcare systems. It also strongly predicts employment opportunities, income potential, and job security. In the Norwegian context—where education is relatively accessible but still varies in terms of long-term economic outcomes—educational attainment serves as a proxy for broader SES differences and likely captures many of the social and structural inequalities that influence both mental health trajectories and labour market participation [32]. While additional SES-related variables like income or occupational class could add value, education alone offers a powerful and meaningful lens through which to assess socioeconomic variation in how CoC impacts employment and earnings. Thus, we explored whether education moderates the relationships, i.e., examined whether the interaction effects between the levels of education and continuity of GP care is important.

As seen in Figures E1 and E2 in the Appendix, the predicted employment probabilities overlap across all education categories at low levels of the RGP-CoC indices. However, as RGP-CoC indices values increase, the predicted probabilities decline for the two lower-educated groups (those with low education and high school-level education), while they increase for the two higher-educated groups (those with short and long higher education). Around and above the average values of the indices, the probabilities for the lower-educated groups tend to increase slightly, though not significantly. Similarly, Figures E3 and E4 show a comparable pattern for predicted wage income, with a clear divergence by education level. Notably, this pattern of divergence is more pronounced when using the CoC-GP index. The results suggest that continuity of GP care has differential effects on employment and wage outcomes depending on education level. For individuals with higher education, increased continuity is associated with better employment prospects and higher wages. In contrast, for those with lower education, higher continuity does not yield the same benefits and may even be linked to slightly lower employment probabilities at moderate levels of CoC. Higher-educated individuals might be more adept at navigating healthcare systems, communicating effectively with providers, or acting on medical advice—factors that could enhance the effectiveness of ongoing care in supporting labour market attachment. In contrast, lower-educated individuals may face structural or informational barriers that limit their ability to translate continuity into improved work outcomes, particularly if other social determinants (e.g., job precarity, poor working conditions) are at play. This divergence is more pronounced when using the CoC-GP index, which may better capture systemic or provider-level aspects of continuity rather than the individual patient–provider relationship.

Figures E5 and E6 illustrate how education level moderates the relationship between CoC indices and the predicted probability of sickness absence. As RGP-CoC indicies increase, predicted probabilities of sickness absence rise for the two higher-educated groups, while remaining relatively stable for the lower-educated individuals. Around and above the average values of the UPC index, sickness absence probabilities tend to decline across all education groups; however, they remain significantly higher for the higher-educated groups. In contrast, for the CoC-GP index, sickness absence probabilities appear relatively constant across all levels for the lower-educated groups. These findings suggest that the relationship between continuity of GP care and sickness absence is shaped by educational attainment. Among higher-educated individuals, increased continuity—particularly as measured by the UPC index—is associated with greater likelihood of sickness absence. This may reflect stronger health-seeking behaviour, better access to benefits, or greater awareness of entitlements. For lower-educated individuals, however, continuity of care appears to have little influence on sickness absence, possibly due to structural barriers in accessing benefits or differing job conditions that make taking leave more difficult. The flat pattern seen in the CoC-GP index for this group supports the notion that system-level continuity may not be equally impactful across socioeconomic strata.

#### Gender

Studies have consistently found variations in the rates of CMDs between gender. Women tend to have higher rates of depression and anxiety disorders compared to men [1, 33]. The difference in prevalence may be influenced by biological, psychological, and socio-cultural factors [34]. Research also suggests that men and women may have different attitudes towards seeking help for mental health concerns [33]. Men may be less likely to seek professional help due to social norms around masculinity and self-reliance [34, 35]. We examined whether the relationship between continuity of GP care and labour market outcomes is moderated by gender. Figures G1 to G2 present the estimated predicted probabilities of employment and predicted wage income by gender across varying levels of the CoC indices. The results indicate that, irrespective of the index used or its value, there are no significant differences in predicted outcomes between men and women.

Similarly, Figures G3 and G6 display the predicted probabilities of sickness absence by gender across different values of the CoC indices. As the figures show, these probabilities do not differ significantly between men and women across most of the CoC range. However, at the highest values of the CoC-GP index, a slight divergence emerges, with the probability of sickness absence being significantly higher for women than for men. Despite this difference at the upper end of the distribution, the overall pattern suggests that the association between continuity of GP care and labour market outcomes among individuals with CMDs is broadly similar for both genders in the Norwegian context.

## Discussion

In this study, we examined the role of continuity of care with regular general practitioners (RGP-CoC), acknowledging that effective healthcare involves more than technical expertise. It also depends on trust, compassion, and sustained engagement within the patient–provider relationship. Our primary aim was to assess whether continuity of RGP care is associated with labour market outcomes among individuals diagnosed with CMDs. To capture different dimensions of RGP-continuity, we employed two alternative RGP-CoC indices and analysed their associations with employment, wage income, and sickness absence. Furthermore, we explored whether these associations varied by educational attainment and gender, offering insight into potential heterogeneity in the effects across different population subgroups.

The study provides compelling evidence that greater continuity of RGP care is associated with improved labour market outcomes for individuals with CMDs. These associations are more pronounced and statistically robust for wage income than for employment probability, suggesting that RGP-CoC may be particularly important for enhancing the intensive margin of labour market participation—that is, increasing earnings and work capacity among those already employed. The findings also support the hypothesis that better RGP-CoC may facilitate better management of health conditions, thereby reducing the likelihood of sickness absence. Heterogeneity analyses by education further reveal that higher-educated individuals are more likely to engage proactively with healthcare services, with frequent and continuous GP visits supporting early recognition and sustained management of health issues. This may explain their higher rates of sickness absence—due to appropriate diagnosis and access to benefits—as well as better long-term employment outcomes through more effective treatment. In contrast, lower-educated individuals may face greater barriers to help-seeking, such as stigma, limited health literacy, or unstable job conditions that hinder regular GP attendance. Even when care is accessed, the benefits of continuity may be diminished due to fragmented follow-up or less personalized care, reducing its impact on both health and labour market outcomes.

This study is among the first to rigorously examine the relationship between GP continuity of care and labour market outcomes using nationally representative longitudinal data. However, in Australian context, using the UPC index, Sheehan et al. [27] investigated the association between the CoC of GP care and work time loss in patients with low back pain. The CoC score was categorised as high, moderate, or low CoC and they found that higher CoC of GP care is linked to reduced working time loss.

There is a possibility that individuals with severe mental health conditions may tend to seek frequent visits to their RGPs, with whom they share stronger relationships, potentially leading to a higher UPC index. These patients may also be less inclined to participate in the labour market due to the severity of their condition or other factors. The observed association between the UPC index and labour market outcomes could be attributable to the potential endogeneity problem. Another potential issue with the UPC index—a quality of GP care proxy — and assessing the relationship with patient’s labour market performance arises when the number of GP visits with their own RGP is exaggerated due to individuals requiring a doctor’s certificate for long-term sick leave, work assessment allowance, disability pension applications, or other reasons. In such instances, the association between high UPC index and labour market exit may be overestimated. Analogously, individuals with unstable employment or lower wages may be less likely to switch their RGPs if their GP is more lenient in providing sick notes, in particular, consequently resulting in a higher UPC index. Furthermore, as mentioned before, the UPC index may be correlated with unobserved factors that also influence labour market outcomes but were omitted in our analyses. For example, individuals with higher levels of social support may exhibit lower UPC indices but experience better labour market outcomes due to these unobserved factors, rather than the continuity of care itself. Therefore, the observed association between the UPC index and better labour market outcomes might then stem from selection bias. Using individual- and GP-level fixed effects, along with controls for individual health status, helps mitigate selection bias to some extent, though it may not fully eliminate it. While we observed similar associations with labour market outcomes for both RGP-CoC indices—with the UPC index showing a relatively higher magnitude—interpreting the absolute size of the associations for the UPC index is more complex. This complexity arises because the UPC index is based on the number of visits an individual makes to their RGP, making it more susceptible to reverse causality and health-related selection. In contrast, the CoC-GP index is derived from the visit patterns of other patients registered with the same GP, rather than the individual’s own visit history. As such, it is arguably less exposed to individual-level selection bias and may provide a more robust measure of GP-level continuity.

In our analyses both the RGP-CoC indices are based on an extensive longitudinal registry dataset which matched patients with their designated RGPs. Neither our independent nor dependent variables are self-reported, we believe this study lessens both selection bias (attributable to non-response and loss to follow-up) and recall bias, which is a common concern in comparison to studies relying on smaller and self-reported samples. Moreover, by employing a lagged design and accounting for unobserved heterogeneity through high-dimensional fixed effects at the individual, GP, and municipality levels, the analysis may mitigate concerns related to endogeneity. This approach strengthens the interpretability of the findings and contributes to the broader understanding of how continuity in GP care may influence economic outcomes.

One of the channels we assumed that the improvement of labour market performance through RGP-CoC occurs via the improvement of health and functioning levels. Using healthcare use from register data as a proxy for health status has the clear advantage of being an objective measure that is much less contaminated by response bias than commonly used survey measures [36]. With this objective and reliable healthcare utilization information we controlled for individuals’ baseline health status and after controlling health status, we found beneficial effects of the continuity of the GP care. This result may indicate that the continuity of GP care, a primary care quality attribute, may not only directly improve CMD-diagnosed patients’ health but also implicitly act to bridge to improve their labour market successes.

Although we controlled for relevant covariates at both the individual and GP levels, and accounting for individual, GP and municipal fixed-effects, our study still has certain limitations. The severity of mental disorders likely affects both the need for continuous care and the capacity to remain in or re-enter the labour market. Including these variables could help identify which subgroups benefit most—or least—from improved RGP-CoC, and would strengthen the policy relevance of the findings by allowing for more targeted interventions. Besides, mental health conditions often arise before age 25 and can disrupt employment trajectories later in life. A Finnish cohort study found serious mental disorders are associated with low employment rates and poor educational outcomes, leading to a substantial loss of total earnings over the life course [37]. Therefore, policies aimed at improving RGP-CoC for younger people with mental health conditions may be especially effective in reducing the long-term economic and social costs of these disorders. Given data limitations, our analysis focused on employment probability, earnings, and sickness absence as key labour market outcomes, as the available registry data did not permit inclusion of additional variables related to job quality and stability. Nonetheless, we acknowledge the potential of comprehensive register data to explore further dimensions such as contract type, employment duration, and occupational mobility in future research.

Moreover, the external validity of the study is limited but informative. Since the research is based on comprehensive Norwegian registry data, the results are highly robust within the Norwegian context, which is characterized by a universal healthcare system, strong primary care infrastructure, and specific labour market regulations. These structural features—especially the gatekeeping role of GPs and generous social welfare policies—may not be directly comparable to those in other high-income European countries with different health system designs (e.g., insurance-based systems in Germany or less centralized GP roles in France or Italy). However, the core mechanism investigated—how continuity of GP care in mental health treatment relates to labour market outcomes—remains broadly relevant across similar contexts. The study provides important insights into the potential value of stable GP-patient relationships for individuals with CMDs, which could inform policy adaptations elsewhere, provided that differences in system design are considered. Future research is needed to replicate and validate these findings in other countries to assess the generalizability of both the associations and the effectiveness of continuity-of-care metrics. Furthermore, interpreting the relationship between RGP-CoC index and labour market outcomes may be complex, as it involves understanding the underlying mechanisms thoroughly. Future studies ought to employ appropriate quasi-experimental econometric techniques, which are imperative for addressing any lasting issues of endogeneity or omitted variable bias (e.g., [38, 39]). This approach will yield more trustworthy estimates of the causal relationship between RGP-CoC and labour market outcomes. In summary, while direct generalization is limited, the study offers a valuable conceptual and methodological framework that can be adapted and tested in other healthcare and labour market settings. Future research that integrates these dimensions could therefore enhance both the explanatory power and external validity of the results.

## Conclusion

Based on our findings, this study concludes that continuity of GP care plays a significant role in shaping labour market outcomes for individuals with common mental disorders (CMDs). Acknowledging potential endogeneity concerns and measurement limitations associated with the UPC index, we employed an alternative CoC-GP index that provides a more conceptually and empirically robust measure of RGP-CoC in relation to labour market outcomes. By addressing endogeneity through a high-dimensional fixed effects model, we found that both RGP-CoC indices demonstrate a consistent positive association with improved labour market outcomes. Overall, our findings highlight the importance of continuity of care as a key factor in shaping primary healthcare services and labour market policies. A well-designed continuity-of-care strategy can not only improve clinical outcomes but also foster economic inclusion and long-term productivity, especially among vulnerable groups such as individuals with mental health conditions. From a policy perspective, we recommend establishing continuity of RGP care as a core quality indicator within primary healthcare systems. Furthermore, efforts should focus on refining and standardizing continuity metrics to enable policymakers and healthcare professionals to more effectively monitor, evaluate, and incentivize continuity within care systems. Finally, future research should continue to develop innovative methods for measuring continuity of RGP care across diverse patient populations and healthcare settings. Such work will be vital to validating continuity as a meaningful indicator of both healthcare quality and broader social outcomes.

## Supplementary Information

Below is the link to the electronic supplementary material.

Supplementary Material 1 (DOCX 323 KB) 

## Data Availability

The data analyses are based on administrative register data stored on a secure server at the University of Bergen, Norway. Due to legal and ethical restrictions, the data are available only to a limited group of researchers and cannot be shared publicly. Researchers may apply for access through the appropriate data providers, subject to approval and compliance with applicable regulations.

## Footnotes

1 The concept of CoC is wide-ranging, but most often refers to personal continuity, either measured longitudinally or by standardized questionnaires eliciting information on patient–provider relational continuity. Informational continuity and management continuity are used as measures of level of seamless services across different care providers or sites [8, 9].

2 We use the Stata module *reghdfe* to estimate the models. To ensure proper inferences and to improve computational efficiency in our high-dimensional fixed-effect regressions we follow Correia [31]and drop singleton observations.
