# Supplementary material for: Bridging success in the labour market: does continuity of general practitioners’ care matter for individuals with common mental disorders?
Source: Eur J Health Econ. 2026 Jan 7;27(6):1277–91. doi: 10.1007/s10198-025-01882-4 (PMC13451221; doi:10.1007/s10198-025-01882-4)
Supplement: Supplementary file 1 [file 10198_2025_1882_MOESM1_ESM.docx]

**Appendix A:**

Table A1: Detail Estimation Results from Fixed-Effects Models: Associations Between CoC Indices and Labour Market Outcomes (Employment Probability, Wage Income, and Sickness absence probability)

| Variable | **Dependent variable:**  **Employment probability** | | **Dependent variable:**  **Log of Wage Income** | | **Dependent variable:**  **Sickness Absence probability** | |
| --- | --- | --- | --- | --- | --- | --- |
|  | **UPC Index** | **CoC-GP index** | **UPC Index** | **CoC-GP index** | **UPC Index** | **CoC-GP index** |
|  | Coefficient  (Robust std. err.) | Coefficient  (Robust std. err.) | Coefficient  Robust std. err. | Coefficient  Robust std. err. | Coefficient  Robust std. err. | Coefficient  Robust std. err. |
| Linear CoC | -0.022^**^  (0.011) | 0.040  (0.042) | 0.208  (0.086) | 0.411  (0.157) | -0.277**^***^**  (0.038) | -0.301**^***^**  (0.089) |
| Quadratic (CoC^2^) | 0.015^*^  (0.009) | -0.115  (0.077) | -0.858  (0.191) | -1.182  (0.339) | 0.879**^***^**  (0.092) | 0.798**^***^**  (0.169) |
| Cubic (CoC^3^) | --- | 0.080^*^  (0.045) | 0.648  (0.119) | 0.828  (0.211) | -0.632**^***^**  (0.057) | -0.540**^***^**  (0.099) |
| **Average marginal effect (AME) of CoC** | **0.003**  **(0.005)** | **0.021^*^**  **(0.012)** | **0.314^***^**  **(0.045)** | **0.232^***^**  **(0.054)** | **-0.307^***^**  **(0.019)** | **-0.139^***^**  **(0.023)** |
| Specialist physical care (spe_Phy) | -0.006^***^  (0.001) | -0.006^***^  (0.001) | -0.034^***^  (0.004) | -0.034^***^  (0.004) | 0.024^***^  (0.002) | 0.024^***^  (0.002) |
| Specialist phycological care (Spe_Psy) | -0.020  (0.019) | -0.020  (0.019) | -0.015  (0.069) | -0.017  (0.069) | -0.047^**^  (0.018) | -0.049^**^  (0.019) |
| Education (Educ1: omitted category) | | | | | | |
| High School Education (Educ2) | 0.011  (0.008) | 0.011  (0.008) | 0.033  (0.039) | 0.034  (0.039) | 0.057^***^  (0.019) | 0.058^***^  (0.019) |
| Short higher education (Educ3) | 0.077^***^  (0.009) | 0.077^***^  (0.009) | 0.367^***^  (0.051) | 0.367^***^  (0.051) | 0.198^***^  (0.021) | 0.198^***^  (0.021) |
| Long higher education (Educ4) | 0.093^***^  (0.011) | 0.093^***^  (0.011) | 0.485^***^  (0.074) | 0.484^***^  (0.074) | 0.265^***^  (0.029) | 0.265^***^  (0.029) |
| GP practicing in a group (GP_group) | -0.011  (0.008) | -0.011  (0.008) | -0.044  (0.037) | -0.048  (0.038) | 0.007  (0.023) | 0.005  (0.023) |
| GP has open list to take new patient (GP_open) | -0.001  (0.001) | -0.001  (0.001) | -0.005  (0.008) | -0.005  (0.008) | 0.001  (0.003) | 0.001  (0.003) |
| Number of observations | N= 371,825 | N=371,825 | N=269,680 | N=269,680 | N= 371,825 | N=371,825 |
| Workers fixed effects | Yes  (n= 139,873) | Yes  (n= 139,873) | Yes  (n= 105,800) | Yes  (n= 105,800) | Yes  (n= 139,873) | Yes  (n= 139,873) |
| GP fixed effects | Yes  (n=2,683) | Yes  (n=2,683) | Yes  (n=2,588) | Yes  (n=2,588) | Yes  (n=2,683) | Yes  (n=2,683) |
| Municipality Fixed effect | Yes  (n=422) | Yes  (n=422) | Yes  (n=420) | Yes  (n=420) | Yes  (n=422) | Yes  (n=422) |
| Time fixed effects | Yes  (n=4) | Yes  (n=4) | Yes  (n=4) | Yes  (n=4) | Yes  (n=4) | Yes  (n=4) |
| Adjusted R2 | 0.762 | 0.762 | 0.689 | 0.689 | 0.511 | 0.511 |

**Appendix: B Figures**

Figure E1: Predicted employment probability by education and UPC index

Figure E2: Predicted employment probability by education and CoC-GP index

Figure E3: : Predicted wage income by education and UPC index

Figure E4: : Predicted wage income by education and GP-CoC index

Figure E5: Predicted sickness absence probability by education and UPC index

Figure E6: Predicted sickness absence probability by education and GP-CoC index

Figure G1: Predicted employment probability by gender and UPC index

Figure G2: Predicted employment probability by gender and Coc-GP index

Figure G3: Predicted wage income by gender and UPC index

Figure G4: Predicted wage income by gender and Coc-GP index

Figure G5: Predicted sickness absence probability by gender and UPC index

Figure G6: Predicted sickness absence probability by gender and CoC-GP index
